# Author reply to Letter to the Editor regarding ‘Understanding subjective experience in psychedelic-assisted psychotherapy: The need for phenomenology’

**DOI:** 10.1177/00048674231211829

**Published:** 2023-11-05

**Authors:** Riccardo Miceli McMillan, Anthony Vincent Fernandez

**Affiliations:** 1 Faculty of Medicine, Mayne Medical School, University of Queensland, Herston, QLD, Australia; 2 Faculty of Arts and Education, Deakin University, Burwood, VIC, Australia; 3 Department of Sports Science and Clinical Biomechanics, University of Southern Denmark, Odense, Denmark

To the Editor

We would like to thank Bianca Ventura for critically engaging with our recent article, ‘Understanding subjective experience in psychedelic-assisted psychotherapy: The need for phenomenology’. As we have read her response, Ventura argues that phenomenology alone is not enough to adequately understand the mechanisms of psychedelic-assisted psychotherapy. She suggests that our proposal be incorporated into a broader theoretical and methodological framework that integrates first-person and third-person data—such as neurophenomenology or non-reductive neurophilosophy (NRNP). As she argues, ‘phenomenology alone remains blind to the third-person perspective of empirical research’ (Ventura, 2023). Approaches like neurophenomenology and NRNP aim to address this shortcoming, providing ways of bridging the phenomenological focus on subjective experience with more traditional ways of investigating the mind and brain.

In response to Ventura, we first want to clarify that we are not opposed to the aims of neurophenomenology or NRNP. On the contrary, we acknowledge the general utility and value that come from employing theoretical and methodological frameworks that integrate phenomenology with other approaches in the cognitive and neurosciences (for more on the promises of neurophenomenology in psychedelic research, see Houot, 2021). A comprehensive understanding of psychedelics—including psychedelic-assisted psychotherapy—will certainly require a diversity of disciplinary perspectives, which will need to be integrated through novel theories and methods. Our aim in the article, however, is not to establish a grand unifying theory that brings all of this together. Our intention is rather to establish a framework for studying subjective experiences in psychedelic-assisted psychotherapy in a comprehensive and nuanced way. We believe that despite theoretical advances on how to integrate first-person and third-person data, we still lack effective tools for generating and analysing the kind of first-person data that we require. Considering this, how to investigate subjective experience is—for us—a more pressing question than how to integrate these investigations into a broader scientific research program.

The primary aim of the research program we propose is to systematically map out subjective alterations in psychedelic-assisted psychotherapy, such as alterations in affective, intersubjective, spatial, temporal, embodied, and self-experiences. This is, first and foremost, a descriptive project. Its value for other research programs is open and, as of yet, undetermined. If adherents of NRNP want to use these phenomenological descriptions for their own aims (e.g. to correlate psychedelic experiences with neurological states), they should certainly do so. But there are plenty of other research programs that might be informed or advanced by knowledge about these subjective alterations. One might, for instance, correlate specific subjective states with reported changes in well-being, or with long-term risks of suicidality. This kind of research could be of immense value to clinical practice—but can likely proceed without any significant theoretical advances on how to integrate first-person and third-person data.

In recent years, it has become abundantly clear that systematic investigations of subjective experience will be an essential component to the success of psychiatry—in terms of both research and clinical practice (Kyzar and Denfield, 2023). The research program we have proposed aims to characterise psychedelic experiences comprehensively and systematically by drawing on phenomenology’s conceptual and methodological resources. Insofar as the project is primarily descriptive, its metaphysical and theoretical neutrality is—in our opinion—a feature rather than a flaw. By focusing on the need for understanding subjective experiences, we have proposed a project that can inform a wide variety of psychiatric research programs, regardless of their theoretical commitments.

## References

[bibr1-00048674231211829] Houot AM (2021) Phenomenology for psychedelic researchers: A review of current methods & practices. Journal of Consciousness Exploration and Research 12: 224–241.

[bibr2-00048674231211829] Kyzar EJ Denfield GH (2023) Taking subjectivity seriously: Towards a unification of phenomenology, psychiatry, and neuroscience. Molecular Psychiatry 28: 10–16. 36460728 10.1038/s41380-022-01891-2 PMC10130907

[bibr3-00048674231211829] VenturaB (2023) Letter to the Editor regarding “Understanding subjective experience in psychedelic-assisted psychotherapy: The need for phenomenology”. Australia and New Zealand Journal of Psychiatry 58: 92–93.10.1177/0004867423120039237767966

